# Challenging suicide, burnout, and depression among veterinary practitioners and students: text mining and topics modelling analysis of the scientific literature

**DOI:** 10.1186/s12917-021-03000-x

**Published:** 2021-09-06

**Authors:** Marta Brscic, Barbara Contiero, Alessandro Schianchi, Cristina Marogna

**Affiliations:** 1 Department of Animal Medicine, Production and Health (MAPS), University of Padova, Agripolis - Viale dell’Università 16, 35020 Legnaro, PD Italy; 2 Fornovo di Taro, Italy; 3 Department of Philosophy, Sociology, Education and Applied Psychology (FISPPA), University of Padova, Piazza Capitaniato 3, 35139 Padova, PD Italy

**Keywords:** Compassion fatigue, Euthanasia, Psychological wellbeing, Team support, Veterinarian, Veterinary student

## Abstract

**Background:**

Worldwide, veterinary practitioners and students are reported to be at higher risk of suicide, burnout, and depression compared to other occupational groups. The aim of the current study was to apply text mining and topic modelling analysis on scientific literature regarding suicide, burnout, and depression among veterinary practitioners and students to extract meaningful and synthetic information. These statistical approaches can be used to comprehend more in deep the phenomena involving veterinarians and veterinary students and to suggest the potential changes needed in admission to veterinary school, veterinary curricula, and post-graduation initiatives as preventive actions.

**Results:**

A systematic search protocol was set up to identify scientific literature that published on the topic from 1985 to 2019. Two-hundred-eleven records were selected with abstracts/texts submitted to text mining and topic modelling analysis. *Student*, *stress*, *work*, anim*, and *euthanasia* resulted the most frequent terms. Topics modelling allowed to differentiate groups of words and papers in 3 areas of interest: 1) students’ difficulties encountered during their studies that increase stress and anxiety impairing their psychological health; 2) exposure to death and euthanasia as risk factor for mental health; and 3) need of support among those providing medical and health care, and of supportive group work to cope with such profession.

**Conclusion:**

Based on the most frequent words included in the clouds and on the contents of the papers clusterised in them, some suggestions are interfered. It is emphasized that the veterinary curricula should include courses that prepare them early to deal with animal death and post-death grief of pet owners, to handle ethical dilemmas and moral stressors, to communicate with clients and staff members, to work in team, to balance work-family life and to promote individual and team resources. Specific courses for veterinary practitioners could keep them updated on their new roles and ways to handle them among functioning as potential feedbacks to monitor their psychological wellbeing.

**Supplementary Information:**

The online version contains supplementary material available at 10.1186/s12917-021-03000-x.

## Background

Worldwide, veterinary practitioners and students are reported to be at higher risk of suicide, burnout, and depression compared to other occupational groups [1–4]. According to some of the first papers reviewing the topic, veterinary practitioners committed more suicides due to their attitude towards euthanasia and easy access to drugs for animal euthanasia [5, 6] and some published case studies proof this assumption [7, 8]. Poisoning are the most common mechanism of death among veterinarians [4] consistent with the hypothesis that increased access to lethal drugs may explain a high incidence of suicides. Hence, Crellin and Katz [9] tackle emergency physicians to closely monitor patients exposed to veterinary euthanasia agents who develop central nervous system and respiratory depression, hypothermia, bradycardia, hypotension, or skin injury. However, the frequency of performing animal euthanasia by practitioners explained only 1% of the total variation for depressed mood among other job stressors [10], indicating thus that several different multi-layered private and professional aspects play a role as suggested by Dilly et al. [11]. Young and female veterinarians are at greatest risk of negative outcomes such as suicidal thoughts, mental health difficulties, and job dissatisfaction [2]. Main occupational difficulties are related to managerial aspects of the job, long working hours, heavy workload and job demands, poor work-life balance, difficult or challenging interactions with clients, clients’ expectations, and suspected patient/pet abuse by owners [12–14]. These specific reasons linked to attitudes and difficulties encountered by veterinary practitioners could not be straightforwardly related to veterinary undergraduate students. It is likely, therefore, that different stressors influence students during veterinary school. According to Reisbig et al. [15], both academic stress and transitional stress have a relevant impact on veterinary students’ well-being in the areas of anxiety and depression symptoms, life satisfaction, general health, perception of academic performance, and grade point average. Findings by Drake et al. [16] pointed out elevated scores of anxiety and depression, particularly high for students in their second and third years of veterinary school. It is reported that factors related to perceived physical health, unclear expectations, difficulty fitting in, heavy workload, and homesickness were most relevant in explaining anxiety and depression symptom prevalence. According to Hafen et al. [17], high relationship quality can have a positive safeguard effect on veterinary students’ depressive symptoms, lower stress associated with balancing their school and home lives, less relationship conflict, better physical health, and improved ability to cope with academic expectations, while at the same time experiencing more stress from being behind in studies.

Low self-confidence in the own competences and doubts about the value of the own work were suggested as further factors affecting negatively veterinarian’s mental health [18], along with higher empathy with animals and compassion fatigue as suggested by some authors [19, 20]. Thus, attitudes toward animals and animal suffering/welfare are investigated in both, veterinary undergraduate students and in veterinary practitioners [21]. The changing nature of animal-human relationships, the emerging trends in human society towards diversification and alternative lifestyles [22], and the debate on the role of veterinarians in recognizing and intervening in the cycle of animal abuse and interpersonal domestic violence [13, 23, 24] seem further raising expectations from veterinarians. On the contrary, it has not been proven that veterinarians were exposed themselves to greater levels of traumatic events in their childhood compared to people involved in other professions [25].

Main limitations of the published studies on the topic of veterinary suicide, burnout, and depression, however, are the distortion of the distribution of the sample due to the self-selection effect of participants who voluntarily take part to cohort studies thus it depends on the instruments used to reach veterinary staff and students or linked to the case studies. It was aim of the current study to apply a different methodology, namely text mining analysis and topic modelling, to extract meaningful numeric indices from unstructured data gathered from a systematic literature review on suicide, burnout, and depression among veterinary practitioners and students considering in particular their mental state. This statistical approach can be helpful to represent the state of the art of scientific knowledge regarding this phenomenon in the perspective of pointing out the changes needed in the veterinary curricula, admission to veterinary school, or post-graduate specific initiatives.

## Results

**Fig. 1:**
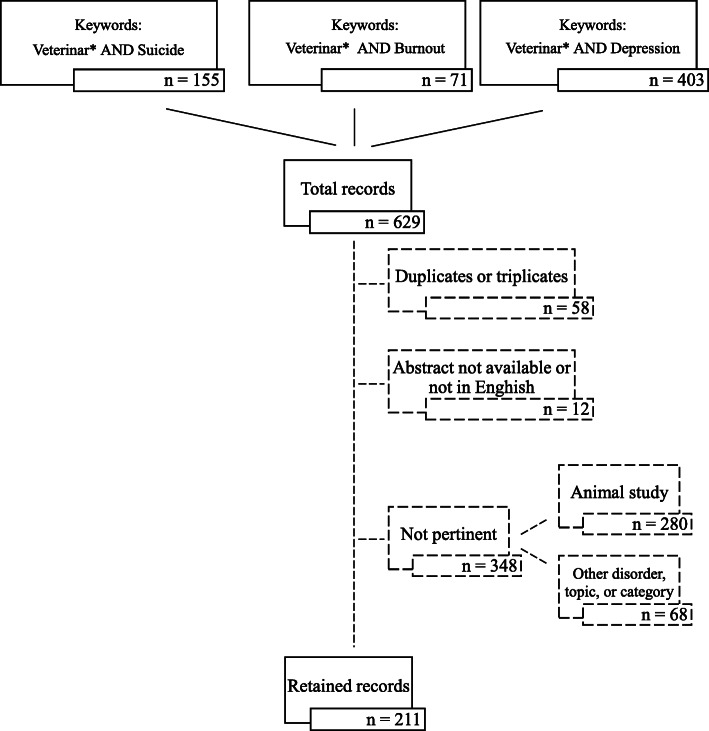
Flow chart representing scientific literature search and numbers of records resulting from each search set. Legend: Reasons for exclusion and number of records excluded from the study after manual screening of the full literature search are represented by the dashed lines.

**Fig. 2:**
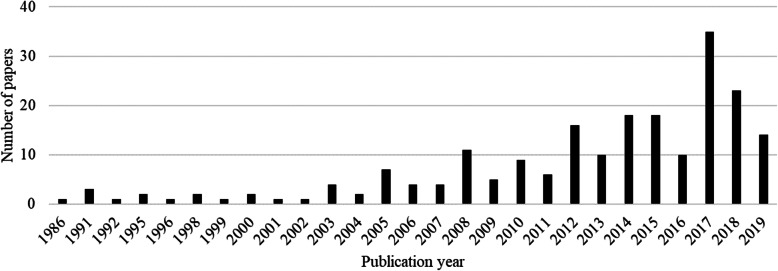
Distribution of number of records per publication year. Legend: Results for year 2019 are related to the period from January to June.

Results of the systematic scientific literature search and the manual screening of papers represented schematically in Fig. 1, produced a total of 211 retained papers. Descriptive statistics showed that they were published in 74 Scientific Journals and one proceeding. The main journal titles publishing on suicide, burnout, and depression among veterinary practitioners and students are listed in Table 1 with 56.4% of records published in these 6 Journals. The remaining journals published a single paper (*n* = 52), 2 (*n* = 12), 3 (*n* = 3), or 4 (*n* = 2) papers on the topic. Journal scientific and subject areas with the total number of journals per area are reported in Table 2. Journal ranking showed that 21 journals with the highest ranking (first quartile = Q1) is within the Medicine area followed by Veterinary (Table 3). The trend over time of the number of papers published on the topic is shown in Fig. 2 with a peak during year 2017.

**Table 1:** The main journal titles publishing on the topic of interest with at least 5 records/each

Journal title	Number of records
Journal of Veterinary Medical Education	48
Javma - Journal of the American Veterinary Medical Association	25
Veterinary Record	25
Australian Veterinary Journal	8
Canadian Veterinary Journal - Revue Veterinaire Canadienne	8
Irish Veterinary Journal	5

**Table 2:** Number of journals per scientific macro and subject area publishing on the topic of interest

Scientific macro area	Subject area	Number
Agricultural and Biological Sciences	Agricultural and Biological Sciences (miscellaneous)	1
	Animal Science and Zoology	5
	Ecology, Evolution, Behavior and Systematics	1
Arts and Humanities	Arts and Humanities (miscellaneous)	3
Biochemistry, Genetics and Molecular Biology	Biochemistry, Genetics and Molecular Biology (miscellaneous)	1
	Genetics	2
Business, Management and Accounting	Organizational Behaviour and Human Resource Management	1
Chemical Engineering	Chemical Health and Safety	1
Chemistry	Analytical Chemistry	1
Computer Science	Computer Science (miscellaneous)	1
Environmental Science	Environmental Chemistry	1
	Health, Toxicology and Mutagenesis	3
Health Professions	Health Information Management	1
Medicine	Medicine (miscellaneous)	24
	Anatomy	1
	Emergency Medicine	1
	Epidemiology	3
	Health Policy	1
	Pathology and Forensic Medicine	3
	Psychiatry and Mental Health	11
	Public Health, Environmental and Occupational Health	8
Nursing	Issues, Ethics and Legal Aspects	1
Pharmacology, Toxicology and Pharmaceutics	Pharmacology	2
	Toxicology	3
Psychology	Psychology (miscellaneous)	2
	Applied Psychology	5
	Clinical Psychology	5
	Developmental and Educational Psychology	1
	Experimental and Cognitive Psychology	1
	Social Psychology	4
Social science	Social Science (miscellaneous)	1
	Anthropology	2
	Education	7
	E-learning	1
	Health (social science)	3
	Sociology and Political Science	3
Veterinary	Veterinary (miscellaneous)	24
	Food Animals	1
	Equine	1
	Small Animals	2
Not classified		5

**Table 3:** Number of journals publishing on the topic of interest according to their scientific macro-area rankings

Scientific macro area	Number of Journals with rank^a^			
	Q1	Q2	Q3	Q4
Agricultural and Biological Sciences	3	1	0	1
Medicine	21	7	1	2
Psychology	9	2	0	2
Social science	11	1	1	0
Veterinary	12	6	6	1
Other^b^	7	1	1	0

^a^The journal was attributed to the scientific macro area according to its highest ranking or to both or more in case of equal rankings in two or more areas

^b^Other includes Arts and Humanities, Biochemistry, Genetics and Molecular Biology, Business, Management and Accounting, Chemical Engineering, Chemistry, Computer Science, Environmental Science, Health Professions, Nursing, Pharmacology, Toxicology and Pharmaceutics with ≤5 Journals each

**Fig. 3:**
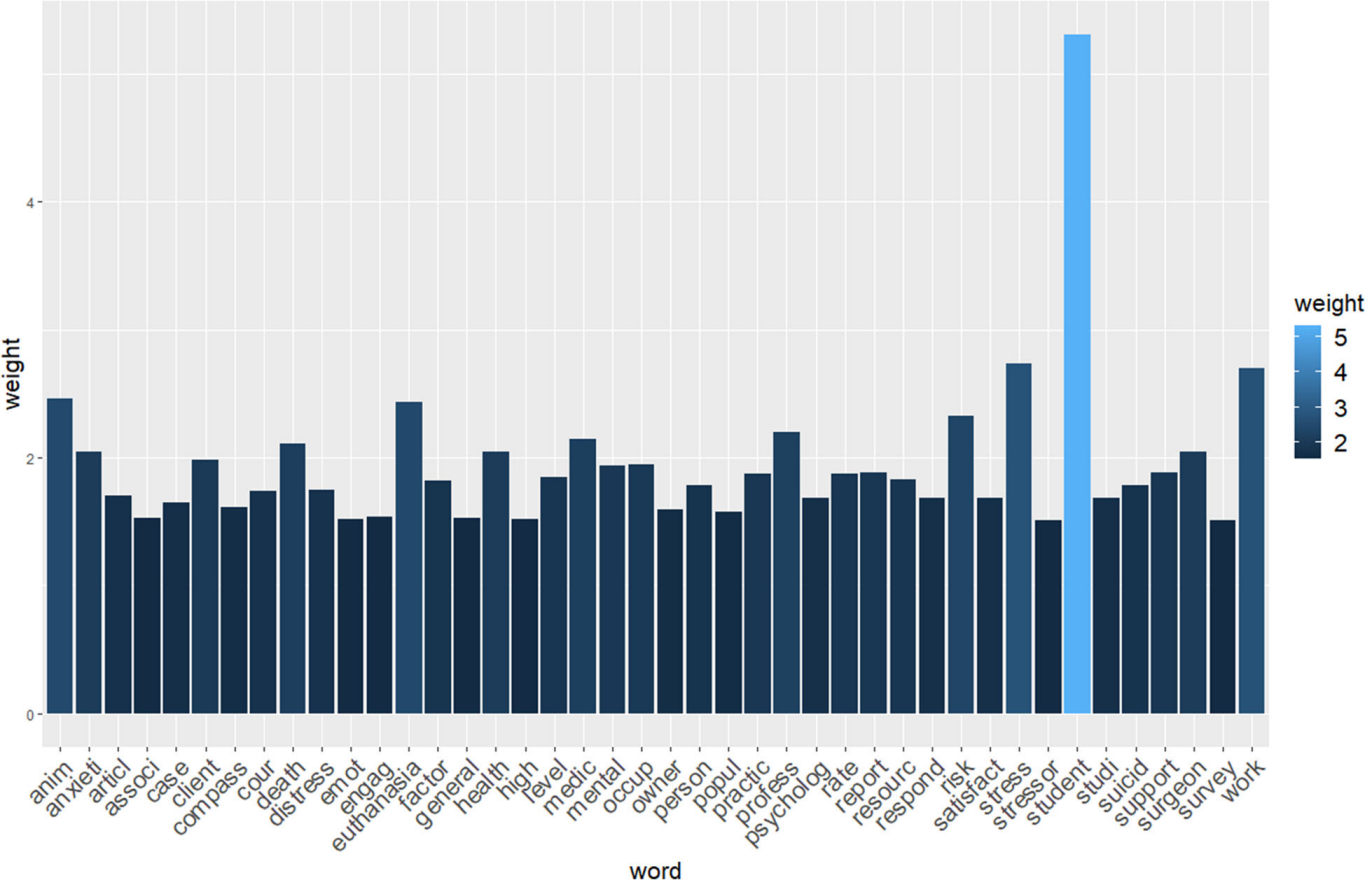
Histogram with the words that have the greatest impact and their respective weights

Text mining analysis on the retained records (179 abstracts and 32 texts), produced a document term matrix with 211 rows (1 row per record) and 3631 columns (1 column per word root) and, after removal of sparse words, the total number of word roots resulted in 1352. Their weights (calculated as TFIDF) ranged from 5.29 for *student* to 0.02 for *allevi*. The most frequent words and their respective weights are reported in the histogram shown in Fig. 3 and *student* appears to be the most frequent word. In addition to the word ‘student’, the terms with weights > 2.0 were: stress, work, anim*, euthanasia, risk, medic*, profess, death, surgeon, health, anxiety.

**Fig. 4:**
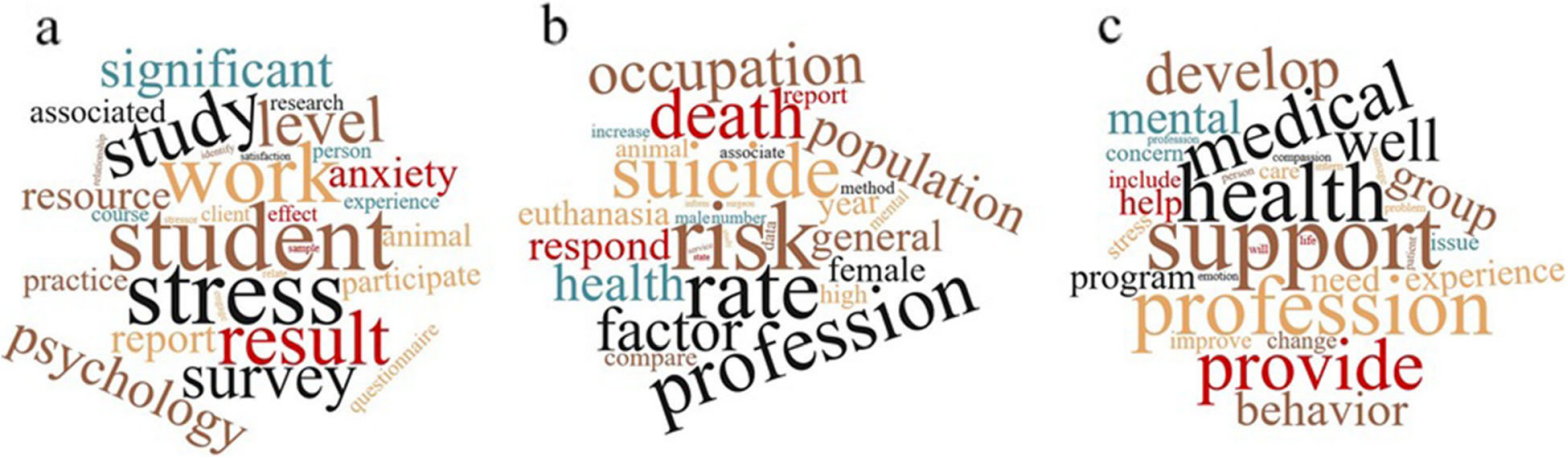
Word clouds with terms grouped by models with 3 topics representing the 3 different areas

Once, the most frequent words and their weights were extracted, the dataset was submitted to topic modelling in order to cluster words and the papers containing them to extract meaningful information. The optimum number of groups for the topic analysis was calculated based on the measures of log-likelihood and perplexity of models fitted with different number of topics but it didn’t provide clear indication because the values of two statistics were continually decreasing with the increase of the number of groups (results not shown). No local minimum was found in the two functions, thus, arbitrarily the re-grouping of words put together by the topic modelling with 3 topics allowed a good distinction of three main areas that are represented in Fig. 4. One area puts together 56 publications and the words related to students’ difficulties encountered during their studies that increase stress and anxiety impairing their psychological health; another area groups 90 papers and their contents underline exposure to death and euthanasia as a risk factor for mental health by profession; and another area groups 65 papers with words that advocate for the need of support among those providing medical and health care, and of group work in order to cope with such profession. Several papers deal with topics that cover more than one area, but they were statistically assigned to one of the three.

## Discussion

Publications on the topic of suicide, burnout, and depression among veterinary practitioners and students are either case studies, cohort or cross-sectional studies based on the voluntary response of respondents, or reviews. In this study we applied text mining analysis and topic modelling in order to extract meaningful numeric indices from unstructured data gathered from a systematic literature review as previously done for other topics [26]. We extracted the most relevant words related to this topic from the published literature and through the clustering of the papers and reviewing their contents we suggested potential changes needed in the veterinary curricula or in the admission to veterinary school for undergraduates and specific post-graduation initiatives needed for practitioners to prevent this issue. Our results revealed a wide variety of subject areas interested in publishing on suicide, burnout, and depression among veterinary practitioners and students. This is a very likely indication of a multidisciplinary interest in this topic and from different perspectives. The medical macro area seems the main interested one with several publications in high ranking Journals being in their first quartile, although veterinarians are also concerned themselves. Indeed, high rank journals specialized in the publication of topics related to veterinary sciences have given space to publications on suicide, burnout, and depression among veterinary practitioners and students, even though it is not scientifically related to the topics covered in these journals. Whereas, we expected the greatest majority of papers to be published in medical and psychiatric journals in particular, followed by journals of the psychological macro area. Descriptive analysis showed that the number of publications on suicide, burnout, and depression among veterinary practitioners and students has increased over the years [27]. The increasing trend over time of the number of publications could be likely due to a greater openness regarding the topic and less shame about it among veterinary students, teachers, and workers, although it still remains an unsolved problem worldwide. Andriessen [28] argues, however, the need to be cautious in evaluating suicide statistics because they might be underestimated in given countries if the undetermined causes of death are misclassified and this is particularly important if we need data to be internationally comparable. It is likely that the peak of publications on this topic observed in 2017 was due to the number of papers published in the Journal of Veterinary Medical Education (20/35 in 2017) when results of a large survey on the veterinarian wellbeing were published in the USA (Merck Animal Health Veterinarian Wellbeing study) and several studies were carried out also on students and on their predisposition already in veterinary school, before practice. According to the results of the text mining analysis carried out for the purpose of the current paper, *Student* appeared to be by far the most frequent word. On the one hand, the Journal publishing the most on the topic is focused on veterinary students thus it could be expected that it raises the frequency of occurrence of such word. On the other hand, although in a speculative way because we don’t have metanalytic evidence based on response rate, we could reflect on students being more accessible for direct submission of questionnaires and interviews in survey studies compared to veterinary practitioners and, thus, larger numbers of studies related to the topic could involve this category of people. One area resulted from the topic modelling analysis in our study is indeed grouping together publications dealing with students’ difficulties encountered during veterinary studies that increase stress and anxiety impairing their psychological health. An additional explanation, however, raises from an increase of knowledge about the risk of veterinary students developing mental health problems at different levels, an increasing number of them seeking for professional help and different correlated initiatives to support them [16, 17, 29]. When discussing students’ mental health, we could also consider that students with Specific Learning Differences (SpLDs) tend to prefer courses related to academic and professional subjects requiring more practical activities and the veterinary curricula could be attractive to them [30–32], as well as the fact that they could be attracted to veterinary and animal care professions by vocation. Veterinary studies, however, involve a difficult curriculum, long study hours, and a high level of empathy that should be cautiously considered before choosing the study program to avoid drop out and prolonged study years. Additionally, in several countries, students are admitted to veterinary school according to tests that select for high-performance mind sets rather than being targeted to the future professional needs of resilience and self-development in order to cope with a difficult profession. According to Dilly et al. [11] specific measures for active stress reduction acquired during the course could be useful to prevent possible consequences throughout the studies such as substance abuse and could be helpful for preparing students for the future profession counteracting stress during the work placement and subsequent occupation. The same authors suggest the importance of recognizing early the critical eustress threshold before they are exceeded and pass on to severe conditions that might represent a risk for burnout, suicidal thoughts or even lead to suicide.

Another area of the topic modelling analysis in our study is grouping papers advocating for the need of support among those providing medical and health care, and of group work in order to cope with such demanding professions. This is in line with Kimber and Gardner [12] who discuss how high job demands may lead to feelings of emotional exhaustion, cynicism and intention to leave which can negatively affect individuals, teams, organizations, clients and patients. Thus, attention and early recognition of individual and team needs are essential starting points for the psychological wellbeing in the veterinary workplace. As suggested for veterinary nurses by the same authors, effective consultative processes may be needed for all veterinary staff members to identify whether demands are seen as reasonable or excessive, and to collaborate in identifying and implementing solutions for healthier workplaces and reducing staff turnover. They suggest that building solid team member relationships, balancing work-family life and job demands, and promoting individual and team resources should start from professional training and should be reinforced in practice. Supportive group work is important also in relation to the high levels of compassion fatigue reported as part of veterinarians’ workplace-induced stress [19]. A further area of the topic modelling results in our study seem underling indeed that exposure to death and euthanasia are risk factor for mental health in the veterinary profession. Marchitelli [33] explores in details the adverse effects of euthanasia pointing out how traumatic it could be for the animal, the pet owner and the veterinarian whereas in several countries there are no precise guidelines of conduct and codes of practice for veterinarians which exposes them to being cited by difficult clients. Additional deontological issues linked to the profession are the end of life of horses and other animals for which euthanasia is even more difficult, responsibility for slaughter and farm audits for animal welfare assessment [34], breeding of specific breeds as for example brachicephalic dogs [35] among others that enhance vulnerability of the veterinarians to moral stressors. Trait perfectionism appeared to have a further strengthening role in this [36]. According to Moir and Van den Brink [18] initiatives promoting the wellbeing of veterinarians are urgently needed, because a large number of veterinarians leave the profession far too early. Thus, it would be very important to do more research on veterinary practitioners’ relationships with clients and to investigate about client typology with particular attention to the two extremes: from people who treat animals like humans or like part of their selves not accepting to constrain their animal during a veterinary visit or procedure, having difficulties to overcome their animals’ death and grieving for a long time seeking for support from the veterinarian, to the other extreme of those abusing animals and exhibiting interpersonal violence. This latter is an additional role that veterinarians have worldwide [13], where we could again question whether they are prepared to handle such important responsibility and ethical dilemmas once in practice. In particular, if we add to this the fact that during the last decades an increasing number of animal-rights-activists and vegans enrolled in veterinary school already facing realities of practices on animals that are very different from those that they may accept them to be (e.g. slaughterhouse practices, dehorning of young replacement dairy cattle and other mutilations).

Among the several actions that could be put in place before, during and after veterinary school we could involve in changing the admission test and requirements [37] and interview individuals to investigate their level of empathy with animals and animal welfare orientation (e.g. vegetarian of vegan for ethical reasons, member of animal protection organizations, pro- or against use of animals in science, etc.); prolong the number of years of vet studies or organize specifically the work load and theory to practice ratio [11]; split the curricula in “food producing animals” from “care of animals” in such way not to expose to the animal-origin food production-related operations those who can not handle them but still have potential for being good veterinary practitioners; and introduce psychology modules or alternative specific programs to teach veterinarians to handle future stressors. Emphasis could be put on the needs to introduce in the veterinary curricula courses in which future veterinarians are prepared to deal with animal death and post-death grief of pet owners, to handle ethical dilemmas and moral stressors protecting themselves from a personal psychological point of view, to communicate with clients (farmers, pet owners, slaughterhouse personnel) and team members, and to work in team balancing work-family life. In this regard, promoting positive individual and team resources should start early as part of the studies and promoted later in practice. Pizzolon et al. [38], indeed, showed that workplace strategies that enhance individual and team engagement and mitigate toxic team environments could potentially improve professional quality of life and job satisfaction in veterinary personnel. For graduates it would be essential to organize at University level courses for veterinary practitioners aiming at updating them on the raise of attention towards their mental health and giving them additional instruments to face their new roles and ways to handle them. These courses could be used also as feedback to monitor their commitment to veterinary work (to which level their normal life style and relationships are affected by being a veterinarian), future perspectives and expectations, satisfactions (personal relationships quality and relations to clients), among other potential risk factors for their psychological wellbeing.

## Conclusions

The current paper is result of a systematic scientific literature review to which text mining and topic modelling analysis were applied to find out the most frequent words and to clusterise the papers retrieved from the literature. The later analyses bring novelty to the paper going beyond a review. The descriptive analysis of the publication indexes and scientific macro areas are helpful to represent the state of the art of scientific knowledge regarding this phenomenon and the areas that show greater interest in the topic. The analysis of the clouds of words and the papers clustered in each of them allowed to suggest some changes needed in the veterinary curricula, admission to veterinary school, or post-graduate specific initiatives that are addressed in the published literature.

## Methods

A systematic scientific literature search was carried out on June 24th 2019 on the Web of science – Thomson Reuters™ (WOS) All Databases (Web of Science Core Collection and all Citation Indexes thicked in the search setting) within the entire timespan from year 1985 – present. The first search set used the keywords Veterinar* (the asterisk * is used as a filler for letters within words so Veterinar* stands for Veterinarian, Veterinarians, Veterinary) and *Suicide* in the Topic field with the *AND* boolean operator. It originated 155 records dated from 1986. The second search set used Veterinar* AND Burnout as topic keywords and originated 71 papers dated from 1987. The third search set used Veterinar* AND Depression as topic keywords and originated 403 papers dated from 1990. These search histories were preliminarily exported and saved and titles and abstracts were manually screened for their contents. Fifty-eight papers were excluded because duplicates (51) or triplicates (7) within or among search sets. Papers with abstract not available or not in English (12) were also removed. In order to exclude a large number of papers dealing with topics not pertinent with this study but still carried out within the veterinary field, the remaining records were screened by checking presence in the title or abstract/text of the words listed in Supplementary Table 1S. Thus, 280 records were identified as dealing with animals, animal diseases, animal productions or products or other similar topics. Further 68 records were removed because dealing with studies on veterinary pharmaceutical compounds or medications (drugs), technologies and detection methods, or other topics of which 25 records included misuse of veterinary drugs in human suicide/poisoning (in terms of clinical, toxicological and emergency reports) by humans whom professional category was different from veterinarians, was unknown or on children. The flow chart of exclusion criteria and numbers of papers retained and excluded is schematically represented in Fig. 1.

All type of papers, including research, reviews, reports, and commentary papers (179 abstracts and 32 texts) that were eligible were submitted to descriptive statistics to profile the scientific corpus. An electronic Excel workbook was used to collect the data extracted from these papers as described by Contiero et al. [39]. The spreadsheet was built in a 2-way table format considering every paper (record) as a row and its descriptive information in columns.

The retained 211 papers were submitted to text mining analysis using libraries tm, stringr, and Snowball3 of the statistical R package (R Core Team software, 2020^1^). Text mining analysis was performed to derive patterns and trends from texts to gain a broad understanding of an entire words dataset and to explore its dynamics. This was achieved through the identification of the main words of the data corpus and the study of the word frequency distributions.

As previously done by Contiero et al. [39], several pre-processing steps of text data were performed. Words were converted to lower-case, and stop words, punctuation, brackets, blanks and numerical digits were excluded. In addition, the search sets keywords Veterinar*, *Suicide*, *Burnout*, and *Depression* were also removed from the dataset to avoid poor discriminative information due to their presence in almost all records retrieved. A stemming algorithm was also applied for a reduction of words to their roots (tokenization), to avoid the count of the same word with different grammatical forms (example: “veterinar” is the root of the words veterinary, veterinarian, veterinarians and so on).

A term frequency - inverse document frequency technique (TFIDF) was applied to weight the frequency of a term adjusted for how widely it is used [40]. This approach aims at reflecting how important a given term is in the whole collection of documents. This first text mining step provided infrastructure for constructing a corpus of documents and to transforming it to a document-term matrix which is the input data for the next step, i.e. topics model.

Topics modelling analysis is a tool to uncover the structure of meaningful themes among collections of documents as well as to discover hidden textual patterns [41]. Latent Dirichlet allocation (LDA), one of the most popular approaches to perform topic modelling analysis, was applied to pursue text mining of the corpus of abstracts. A Bayesian probabilistic approach leads to discover a set of thematic topics from words that tend to occur together in a document. A single topic can be described as a multinomial distribution of words, and a single document can be described as a multinomial distribution of latent topics. This model gives both topic representations of all the documents and word distributions of all the topics, in an iterative process implemented using Gibbs sampling. At the end of the iterative process, a posterior distribution was calculated to estimate the topic mixture of each document (by counting the proportion of words assigned to each topic within that document) and the words associated to each topic (by counting the proportion of words assigned to each topic overall).

We used LDA function with Gibbs sampling option of the topic models package in R [42]. The number of topics needs to be fixed a-priori. Because the number is in general not known, models with several different number of topics were fitted and measures of evaluation were calculated (log-likelihood and perplexity). For visualization of topics, the most probable words to covey a topic meaning were listed and represented by clouds (https://www.wordclouds.com/), where the higher colour depth and character size indicate a greater probability. Each document was assigned to a topic with the highest probability.

## Supplementary Information

**Additional file 1.**

## Data Availability

Data sharing is not applicable for this article as the dataset was generated from a scientific literature search using the former Web of science – Thomson Reuters™ database (now Web of Science -© 2020 Clarivate Analytics) and transparently described in the text.

## Footnotes

1 R Core Team. R: A language and environment for statistical computing. R foundation for statistical computing. Vienna, Austria, 2020.
