# Supplementary material for: Challenging suicide, burnout, and depression among veterinary practitioners and students: text mining and topics modelling analysis of the scientific literature
Source: BMC Vet Res. 2021 Sep 6;17:294. doi: 10.1186/s12917-021-03000-x (PMC8419380; doi:10.1186/s12917-021-03000-x)
Supplement: Supplementary file 1 — Additional file 1. [file 12917_2021_3000_MOESM1_ESM.docx]

Caption to Supplementary Table 1S: List of words used to exclude not pertinent records from the literature search.

Supplementary Table 1S

| *Veterinar** and *Suicide* | *Veterinar** and *Depression* |
| --- | --- |
| cellular | alpaca |
| gene | amphibian* |
| genome | apes |
| immunotherap* | avian |
| inbred | baboon |
| inbreeding | bear |
| mutagenesis | bison |
| mutation | bitch* |
| tumor cell | broiler |
|  | buffalo |
|  | canine |
|  | caracaras |
|  | cat |
|  | cattle |
|  | cephalopod* |
|  | cheese |
|  | chemotherapy |
|  | chick* |
|  | chimpanzee* |
|  | cockatiels |
|  | conures |
|  | dandelion* |
|  | deer |
|  | dog |
|  | duck |
|  | eagle |
|  | elephant |
|  | equid* |
|  | equine |
|  | falcon |
|  | feline |
|  | ferret |
|  | fish |
|  | foal |
|  | food |
|  | frog |
|  | frogs |
|  | fungi |
|  | gazelle |
|  | gene |
|  | gerbil |
|  | glinder |
|  | goat |
|  | gorilla |
|  | guinea pig |
|  | hen |
|  | herpetology |
|  | honey |
|  | horse |
|  | iguana |
|  | impala |
|  | inbred |
|  | inbreeding |
|  | insect |
|  | lion |
|  | lizard |
|  | llama* |
|  | macaca |
|  | marine |
|  | mice |
|  | milk |
|  | monkey |
|  | mosquitoe* |
|  | mouse |
|  | mussel |
|  | mutagenesis |
|  | mutation |
|  | ostrich |
|  | otariid |
|  | otter |
|  | panda |
|  | parrot |
|  | penguin* |
|  | pig |
|  | pigeon |
|  | piglet* |
|  | quil |
|  | rabbit |
|  | rainbow |
|  | rat |
|  | rhinocero* |
|  | rodent |
|  | ruminant |
|  | salmon |
|  | seal |
|  | sheep |
|  | shrimp |
|  | suis |
|  | swine |
|  | tiger |
|  | toucan |
|  | trout |
|  | turckey |
|  | turtle |
|  | vaccine |
|  | vector |
|  | viral |
|  | virulence |
|  | virus |
|  | wallaby |
|  | whale* |
|  | wildli* |
|  | wolf |
|  | zoo |

Legend to Supplementary Table 1S: List of words used to exclude from the systematic literature search on the Web of science – Thomson Reuters^TM^ (All Databases (Web of Science Core Collection and all Citation Indexes thicked in the search setting) within the entire timespan from year 1985 to June 2019) records dealing with topics not pertinent with this study but still carried out within the veterinary field using the *NOT* boolean operator in the first search with keywords *Veterinar** and *Suicide* (left) and third search set with keywords *Veterinar** and *Depression.* The search terms with the asterisk were truncated with the symbol * allowing to search all the variants starting with the same root.
